# Identification and validation of autophagy-related genes in primary open-angle glaucoma

**DOI:** 10.1186/s12920-023-01722-5

**Published:** 2023-11-15

**Authors:** Wanjing Xu, Yuhao Sun, Shuang Zhao, Jun Zhao, Juanmei Zhang

**Affiliations:** 1 Ophthalmology Department of QingPu Branch of Zhongshan Hospital Affiliated to Fudan University, Shanghai, China; 2 Otolaryngology Department of QingPu Branch of Zhongshan Hospital Affiliated to Fudan University, Shanghai, China; 3 Graduate School of Shandong First Medical University, Jinan, China; 4 Ophthalmology Department of Linyi People’s Hospital, Linyi, China

**Keywords:** Primary open-angle glaucoma, Autophagy, Differentially expressed genes, Bioinformatics analysis, RT-PCR, HSPA8, RPL15

## Abstract

**Background:**

As the most common type of glaucoma, the etiology of primary open-angle glaucoma (POAG) has not been unified. Autophagy may affect the occurrence and development of POAG, while the specific mechanism and target need to be further explored.

**Methods:**

The GSE27276 dataset from the Gene Expression Omnibus (GEO) database and the autophagy gene set from the GeneCards database were selected to screen differentially expressed autophagy-related genes (DEARGs) of POAG. Hub DEARGs were selected by constructing protein-protein interaction (PPI) networks and utilizing GSE138125 dataset. Subsequently, immune cell infiltration analysis, genome-wide association study (GWAS) analysis, gene set enrichment analysis (GSEA) and other analyses were performed on the hub genes. Eventually, animal experiments were performed to verify the mRNA levels of the hub genes by quantitative real time polymerase chain reaction (qRT-PCR).

**Results:**

A total of 67 DEARGs and 2 hub DEARGs, HSPA8 and RPL15, were selected. The hub genes were closely related to the level of immune cell infiltration. GWAS analysis confirmed that the causative regions of the 2 hub genes in glaucoma were on chromosome 11 and chromosome 3, respectively. GSEA illustrated that pathways enriched for highly expressed HSPA8 and RPL15 contained immunity, autophagy, gene expression and energy metabolism-related pathways. qRT-PCR confirmed that the expression of Hspa8 and Rpl15 in the rat POAG model was consistent with the results of bioinformatics analysis.

**Conclusions:**

This study indicated that HSPA8 and RPL15 may affect the progression of POAG by regulating autophagy and provided new ideas for the pathogenesis and treatment of POAG.

**Supplementary Information:**

The online version contains supplementary material available at 10.1186/s12920-023-01722-5.

## Background

Glaucoma is a chronic, progressive, and irreversible eye disease that can damage the optic nerve and cause characteristic visual field defect. Meanwhile, it is currently the leading cause of irreversible blindness in the world [1, 2]. According to statistics, the number of glaucoma patients worldwide will increase to 111.8 million by 2040 [3], and primary open-angle glaucoma (POAG) is the most common type of glaucoma, accounting for approximately 70% of all cases [4]. The onset of POAG is insidious, characterized by retinal ganglion cell (RGC) death and pitting optic atrophy. Due to the absence of significant subjective symptoms in the early stage, even in developed countries, less than 50% of patients are diagnosed before late irreversible optic nerve injury [5–7]. Therefore, there is an urgent need to improve the screening and treatment of POAG. The pathogenesis of POAG has not been fully understood, and the main risk factor for visual loss is ocular hypertension that is caused by obstruction of aqueous outflow from the trabecular meshwork (TM) -Schlemm’s canal system, although the patient’s anterior chamber angle is open [8].

Autophagy is an intracellular catabolic process, involving the capture and decomposition of various substances through the lysosomal pathway. Moreover, autophagy is activated as an adaptive response to intracellular and extracellular stressors, such as misfolded proteins, damaged organelles, nutrient deprivation, reactive oxygen species, hormones, and infection [9, 10]. The autophagic process not only plays a central role in cell survival, development, differentiation, and homeostasis maintenance, but also has molecular links to the progression of a variety of diseases, including neurodegenerative diseases, cancer, infectious diseases, and immune diseases [9, 11, 12]. As a common neurodegenerative disease, POAG’s pathogenesis may be associated with autophagy. Previous studies have shown that autophagy disorders can lead to ocular diseases, including age-related macular degeneration, cataract, diabetic retinopathy, and thyroid-associated ophthalmopathy [13]. In recent years, the effect of autophagy on POAG has also been gradually revealed, and structural change or dysfunction of TM system as well as accelerated death of RGCs with their axons are important to the pathogenesis of POAG [14, 15]. It has been shown that dysregulation of autophagy triggered progressive functional failure of TM cells and decreased survival rate of RGCs [16–18]. However, the underlying molecular mechanisms of autophagy dysregulation in POAG still need to be further explored.

Bioinformatics is an emerging discipline combining molecular biology and computational science. The collection of diseases’ expression profile data and the use of computational tools for bioinformatics analysis are conducive to understanding the molecular mechanism of diseases and screening biomarkers [19]. In this study, we analyzed a dataset of human POAG and normal individual samples downloaded from the Gene Expression Omnibus (GEO) database, aiming to identify POAG-related differentially expressed genes (DEGs). Afterwards, DEGs were then mapped to the autophagy gene set to obtain differentially expressed autophagy-related genes (DEARGs). Next, Gene Ontology (GO) and Kyoto Encyclopedia of Genes and Genomes (KEGG) [20–22] enrichment analysis and protein-protein interaction (PPI) network analysis were used to elucidate the biological function of DEARGs and select key genes. Another POAG-related dataset was downloaded to verify these key genes and finally 2 hub genes, HSPA8 and RPL15, were obtained. Moreover, the hub genes were validated by quantitative real time polymerase chain reaction (qRT-PCR) in animal experiments, and it was discovered that the expression of the 2 hub genes in the rat chronic ocular hypertension (COH) model was consistent with the bioinformatics analysis results of the human POAG microarray dataset, which suggested that HSPA8 and RPL15 may be involved in the occurrence and development of POAG through regulating autophagy.

## Methods

### Gene expression data acquisition

The GSE27276 data file was downloaded from the GEO database (http://www.ncbi.nlm.nih.gov/geo/) that is annotated by GPL2507 as a Series Matrix File. The file contains data related to 36 groups of patients’ expression profiles, including 19 normal groups and 17 POAG groups. The limma package was used to identify DEGs, the normalizeBetweenArrays function was used to correct batch effects between samples, the lmFit function was used to fit the linear model, and the eBayes function was used to calculate the *P*-values. Meantime, the GSE138125 Series Matrix File was obtained from the GEO database. The annotation platform is GPL21827, which consists of 8 groups of transcriptome data, with 4 cases of normal group and 4 cases of POAG patients, for subsequent validation. The autophagy gene set used in this analysis was downloaded from the GeneCards database (https://www.genecards.org/). Totally, 1929 autophagy-related genes with relevance score > 1 were extracted for this study.

### GO and KEGG functional annotations

To obtain the biological functions and signaling pathways involved in the development of POAG, the Metascape database (https://metascape.org/) was used for annotation and visualization. GO analysis and KEGG pathway analysis were performed on DEGs. Enrichment analyses were carried out according to the *P*-values after correction. Min overlap ≥3 and *P* ≤ 0.01 was considered statistically significant.

### Analysis of immune cell infiltration

In this study, the CIBERSORT algorithm was used to analyze the patients’ data to evaluate the relationship between immune cell types and gene expression. Based on the principle of support vector regression, the algorithm discriminated 22 human immune cell phenotypes, including T cells, B cells, plasma cells and myeloid cell subsets, by deconvolution analysis. Furthermore, Pearson correlation analysis was used to explore the correlation between gene expression and immune cell content.

### Genome-wide association study (GWAS) analysis

The Gene Atlas database (http://geneatlas.roslin.ed.ac.uk/) is a large database that records associations between hundreds of traits and millions of variants using the UK Biobank cohort. These associations were calculated according to 452,264 British individuals in the UK Biobank database, including 778 phenotypes and 30 million loci.

### Gene set enrichment analysis (GSEA)

According to degree of gene differential expression in the two types of samples, GSEA uses predefined gene sets and ranks genes, and then tests whether the prespecified gene set is enriched at the top or the bottom of this ranking table. In this study, the differences in signaling pathways between the high and the low expression group were compared by GSEA to investigate the molecular mechanism of hub genes in the 2 groups of patients. The number of substitutions was set to 1000 and the substitution type was set to phenotype.

### Regulatory network analysis of hub genes

In this study, transcription factors (TFs) were predicted by the R package “RcisTarget”. All calculations performed by RcisTarget were based on motifs. The normalized enrichment score (NES) of the motif depended on the total number of the motif in the database. In addition to the motifs annotated by the source data, further annotation files were deduced based on motif similarity and gene sequences. According to the recovery curve calculation of motif ranking by gene set, the first step in estimating the overexpression of each motif on the gene set was to calculate the area under the curve (AUC) for each pair of motif-motif set. The NES of each motif was calculated from the AUC distribution of all motifs in the gene set. We used “rcistarget .hg19. motifdb. cisbpont .500bp” for the gene-motif rankings database.

### Connectivity map (CMap) drug prediction

CMap, a gene expression profiling database based on intervening gene expression developed by the Broad Institute, is mainly used to reveal the functional links of small molecule compounds, genes, and disease status. Also, it contains gene chip data before and after the treatment of 5 human cell lines with 1309 small molecule drugs. There are various processing conditions, including different drugs, concentrations, treatment durations, and so on. In this study, targeted therapeutic agents were predicted for POAG through DEGs. The results of the analysis would provide each matched drug with the Connectivity Score, which reflected the similarity of the drug to the DEGs. A higher Connectivity Score indicated a higher similarity between the drug and the DEGs. The compound with the lowest negative number was selected as the potential treatment drug for POAG, suggesting that the drug could alleviate or even reverse the POAG disease state.

### Animal and experiment designs

Specific-pathogen free (SPF) male Sprague-Dawley (SD) rats, 6–8 weeks old, weighing about 220 g–240 g, and free of ocular disease under slit-lamp and fundus examination, were provided by Hubei Experimental Animal Research Center (license number: SCXK (Hubei) 2020–0018). The Institutional Research Ethics Committee of Linyi People’s Hospital of Qingdao University fully approved this research (approval number: YX200467). All methods were carried out in accordance with ARRIVE guidelines (https://arriveguidelines.org) for the reporting of animal experiments and Vision Research and the guidelines of Qingdao University on the ethical use of animals. All rats were reared in a temperature- and humidity-controlled room with a 12-hour light-dark cycle. Specialized breeders changed the padding once a day and fed the rats with warm water and laboratory rat food (Medicience Co., Ltd., China). After 1 week of adaptation in the quiet environment, SD rats were randomly divided into COH glaucoma model group and normal control group, and then the rats in the model group were modeled and identified.

Methods of building model: SD rats were anesthetized with intraperitoneal injection of 1% of sodium pentobarbital at 45 mg/kg. The left eye was selected as the operated eye and instilled proparacaine 0.5% eye drops (Alcon, USA). The bulbar conjunctiva was cut lateral to the corneal limbus to separate the subconjunctival fascia and muscles. Three episcleral veins were found near the rectus muscle, respectively, and the common branch of the vein was lifted to be cauterized with a heated pin. Signs of successful cauterization: Venous blood flow disappeared at the cautery site, the proximal vein was congested, and a white line formed in the distal vein. Then the bulbar conjunctiva was sutured and levofloxacin hydrochloride eye gel (Ebe Pharmaceutical Co., Ltd., China) was applied once a day into the conjunctival sac for 5 days after surgery.

Intraocular pressure (IOP) measurement: After anesthesia, the rat cornea was contacted with the tip of the Tono-Pen AVIA tonometer (Reichert, USA), and five consecutive steady readings were recorded for each eye. The mean of the readings was taken as the IOP measurement. IOP was measured before and 30 minutes after surgery. Postoperative IOP was 1.7 times higher than preoperative IOP in the left eye, which was considered as a successful surgery. In this study, 4 rats in the COH glaucoma model group and 4 rats in the normal control group were randomly selected, and IOP was measured weekly until the end of the experiment. Each IOP measurement was performed at 9–10 am by the same person. The cornea and the sclera of the rats were also observed at the same time. Three weeks later, all rats were euthanized with a 10-fold anesthetic dose (450 mg/kg sodium pentobarbital), and ocular tissues from the left eye were taken and stored in a − 80 °C freezer.

### RNA extraction and qRT-PCR

Total RNA was extracted by Trizol (Ambion, USA) from rat ocular tissues weighing approximately 100 mg. Subsequently, OD260, OD280 and OD260/OD280 values were measured by ultraviolet-visible spectrophotometer (MIULAB, China) to calculate the purity and the concentration of the isolated RNA. cDNA was synthesized by HiScript® II Q RT SuperMix for qPCR (+gDNA wiper) (Vazyme, China) using 5 μg RNA. mRNA expression levels of Hspa8 and Rpl15 were quantified using SYBR Green Master Mix (Vazyme, China). The 2^^-ΔΔCt^ method was applied to calculate the relative differences between the model group and the normal control group. Primers were as follows: Rat Gapdh: Forward: 5′-ACAGCAACAGGGTGGTGGAC-3′; Reverse: 5′-TTTGAGGGTGCAGCGAACTT-3′; product length: 253 bp. Rat Hspa8: Forward: 5′-TAGATAAGAAGGTCGGGGCTGAA-3′; Reverse: 5′-TGGGTGCTGGAGGAGAGGGT-3′; product length: 283 bp. Rat Rpl15: Forward: 5′-CCTACTGGGTTGGTGAAG-3′; Reverse: 5′-GTAATCCATTGGGTGTCG-3′; product length: 103 bp.

### Statistical analysis

All statistical analyses were performed by R language (version 4.0) and SPSS (version 22.0). T-test was used for measurement data (expressed as a mean ± standard error). Significance was defined as *P* < 0.05 for two-sided tests.

## Results

### Identification of DEARGs

**Fig. 1:**
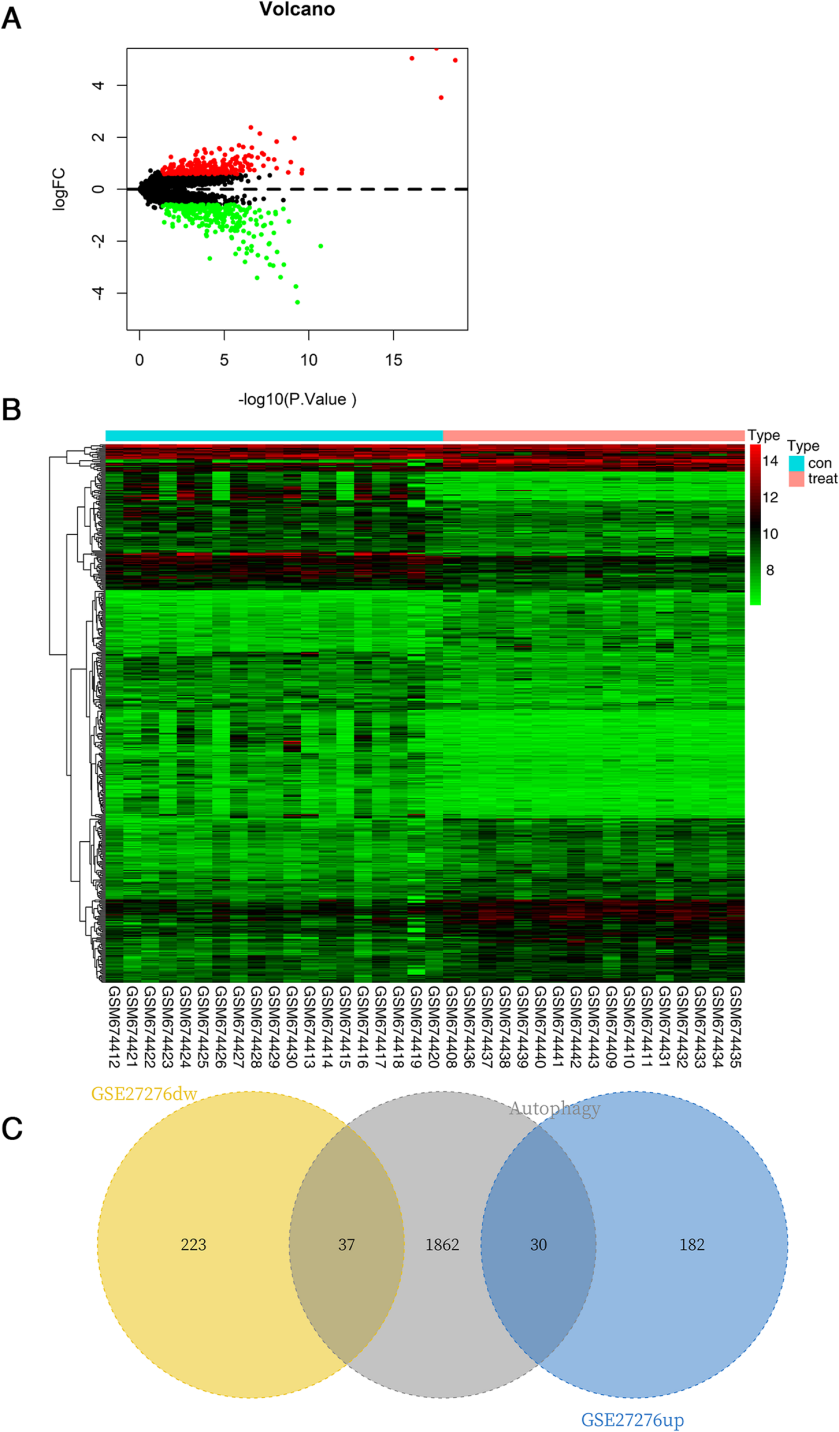
Identification of DEGs and DEARGs. **A** Volcano plot for DEGs between the normal group and the POAG group. The red dots indicated differentially expressed upregulated genes, and the green dots indicated differentially expressed downregulated genes. The screening criteria for DEGs were |logFC| > 0.585 and *P* < 0.05. **B** Heatmap of DEGs analyzed from the dataset of GSE27276. **C** The Venn diagram showed 30 up-regulated DEARGs and 37 down-regulated DEARGs

GSE27276 data file was downloaded from the GEO database for a total of 36 groups of patients, including 19 in the normal group and 17 in the POAG group. The Limma package was used to identify DEGs, and the differential expression threshold was |logFC| > 0.585 and *P* < 0.05. Finally, 472 DEGs (212 upregulated genes and 260 downregulated genes) were detected (Additional files 1, 2 and 3). Volcano plot and heatmap of DEGs were shown (Fig. 1A, B). The DEGs were mapped to the autophagy gene set, thus resulting in 67 DEARGs (30 up-regulated genes and 37 down-regulated genes) (Fig. 1C).

### Enrichment and PPI network analysis of DEARGs and hub gene identification

**Fig. 2:**
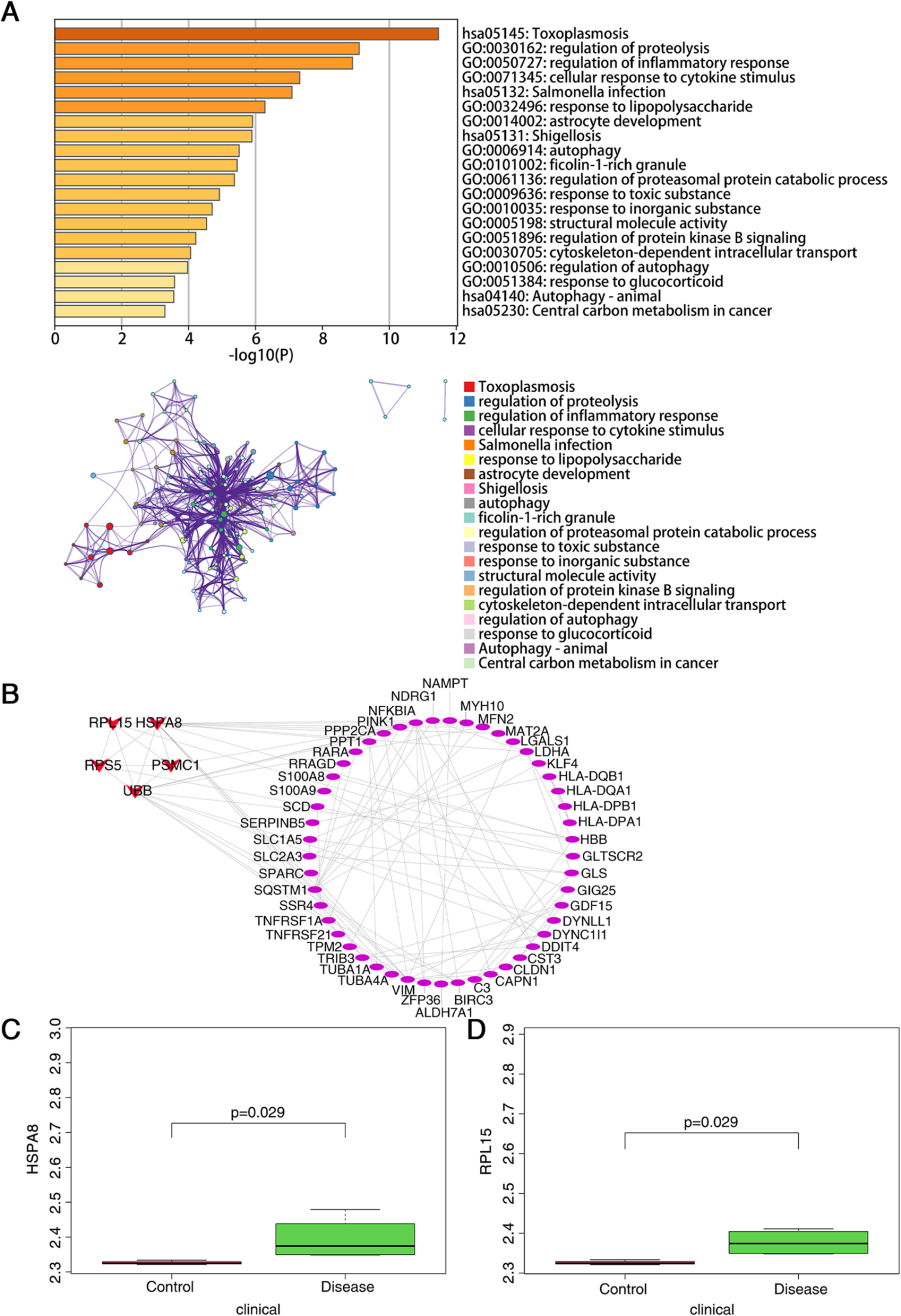
Identification of hub genes. **A** The significant GO terms and KEGG pathways of the DEARGs. **B** The significant modules of DEARGs through PPI network analysis. The PPI network of DEARGs was analyzed by using the STRING database. Medium confidence was set to 0.4, and species was set to human. Color represents connectivity. The higher the connectivity, the darker the color. UBB, RPL15, HSPA8, RPS5 and PSMC1 were selected as key genes. **C**, **D** HSPA8 and RPL15 were identified as the hub genes by external dataset validation

GO and KEGG enrichment analysis were further performed on these 67 DEARGs (Additional file 4). The result proved that DEARGs were mainly enriched in pathways associated with autophagy or glaucoma such as regulation of proteolysis, regulation of inflammatory response, response to lipopolysaccharide, astrocyte development, autophagy, regulation of proteasomal protein catabolic process, regulation of protein kinase B signaling, regulation of autophagy, response to glucocorticoid and autophagy-animal (Fig. 2A). The protein interaction pairs associated with these 67 DEARGs were obtained from the STRING online database [23] and visualized by Cytoscape [24]. According to MCODE (The MCODE algorithm is based on Cytoscape software [24]), the most critical TOP5 genes were selected as key genes, including UBB, RPL15, HSPA8, RPS5 and PSMC1 (Fig. 2B). To further identify hub genes in DEARGs, the GSE138125 dataset of POAG was downloaded from the GEO database for validation. HSPA8 and RPL15 were finally identified as the hub genes of this study (Fig. 2C, D).

### Immune infiltration analyses

**Fig. 3:**
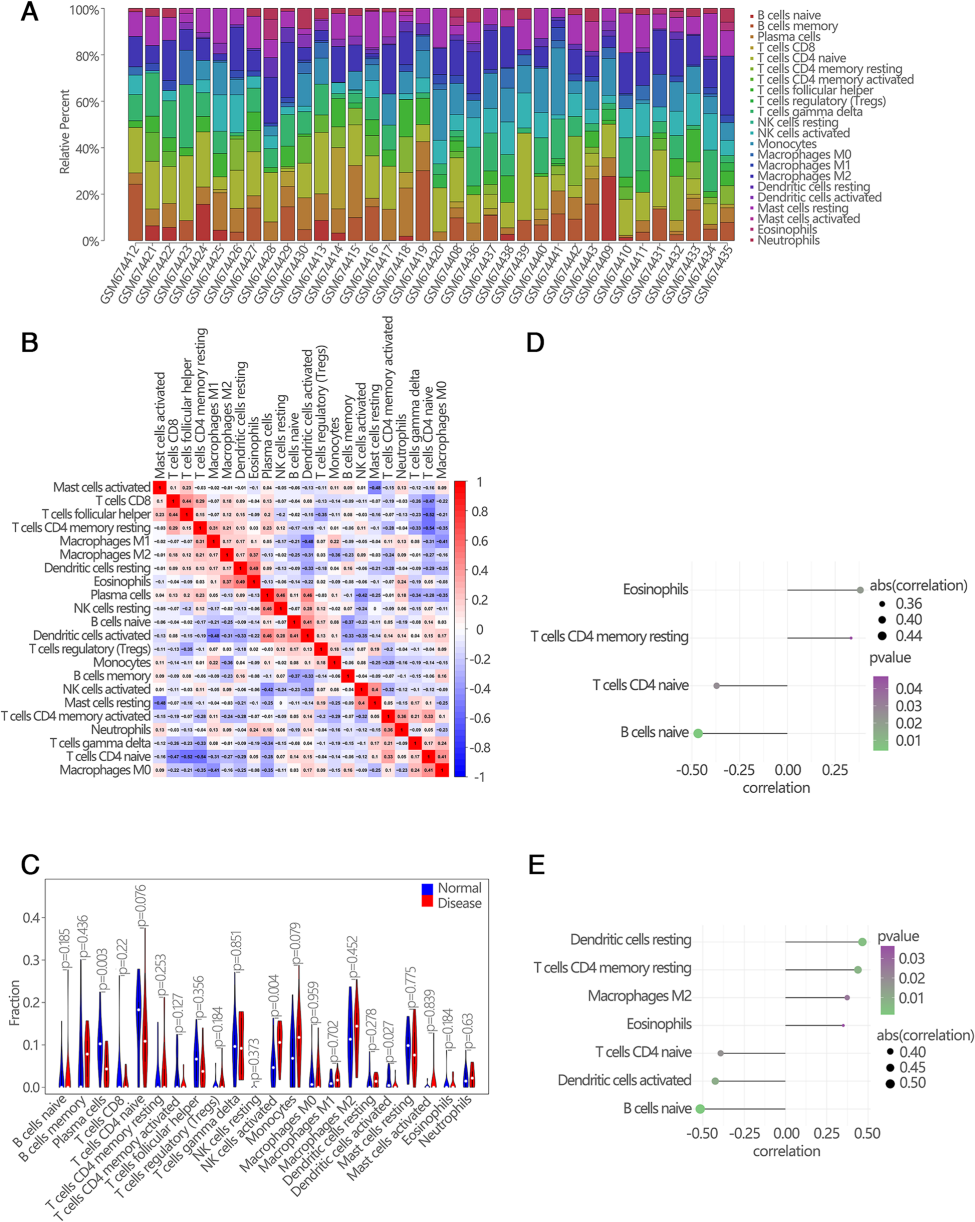
Immune infiltration analyses between POAG groups and normal groups. **A** The relative percentage of 22 subpopulations of immune cells in 36 samples from GSE27276 dataset. **B** The heat map showed the correlation in infiltration of innate immune cells by CIBERSORT. **C** The difference of immune infiltration between POAG groups and normal groups. The normal groups were marked as blue color and the POAG groups were marked as red color. *P* values < 0.05 were considered as statistical significance. **D** Correlation between the immune infiltration level and expression of HSPA8. **E** Correlation between the immune infiltration level and expression of RPL15

The immune microenvironment is mainly composed of immune-related fibroblasts, immune cells, extracellular matrix, a variety of growth factors, inflammatory factors, and special physical and chemical characteristics. The immune microenvironment significantly affects the diagnosis, survival outcome and clinical treatment sensitivity of diseases. The mechanism of hub genes affecting POAG progression was investigated by analyzing the relationship between hub genes and immune infiltration in GSE27276 dataset. The immune cell content of each patient in GSE27276 dataset was shown (Fig. 3A). There were several significant correlation pairs between the levels of immune infiltration (Fig. 3B). Plasma cells and Dendritic cells activated significantly lowered in POAG groups compared with normal groups (Fig. 3C). The relationship between hub genes and immune cells was investigated and it was found that hub genes were highly correlated with immune cells. HSPA8 was positively correlated with Eosinophils and T cells CD4 memory resting, while negatively correlated with B cells naive and T cells CD4 naive (Fig. 3D). RPL15 was positively correlated with Dendritic cells resting, T cells CD4 memory resting, Macrophages M2 and Eosinophils, while negatively correlated with B cells naive, Dendritic cells activated and T cells CD4 naive (Fig. 3E). These analyses confirmed that hub genes were closely related to the level of immune cell infiltration and played an important role in the immune microenvironment.

### GWAS analysis

**Fig. 4:**
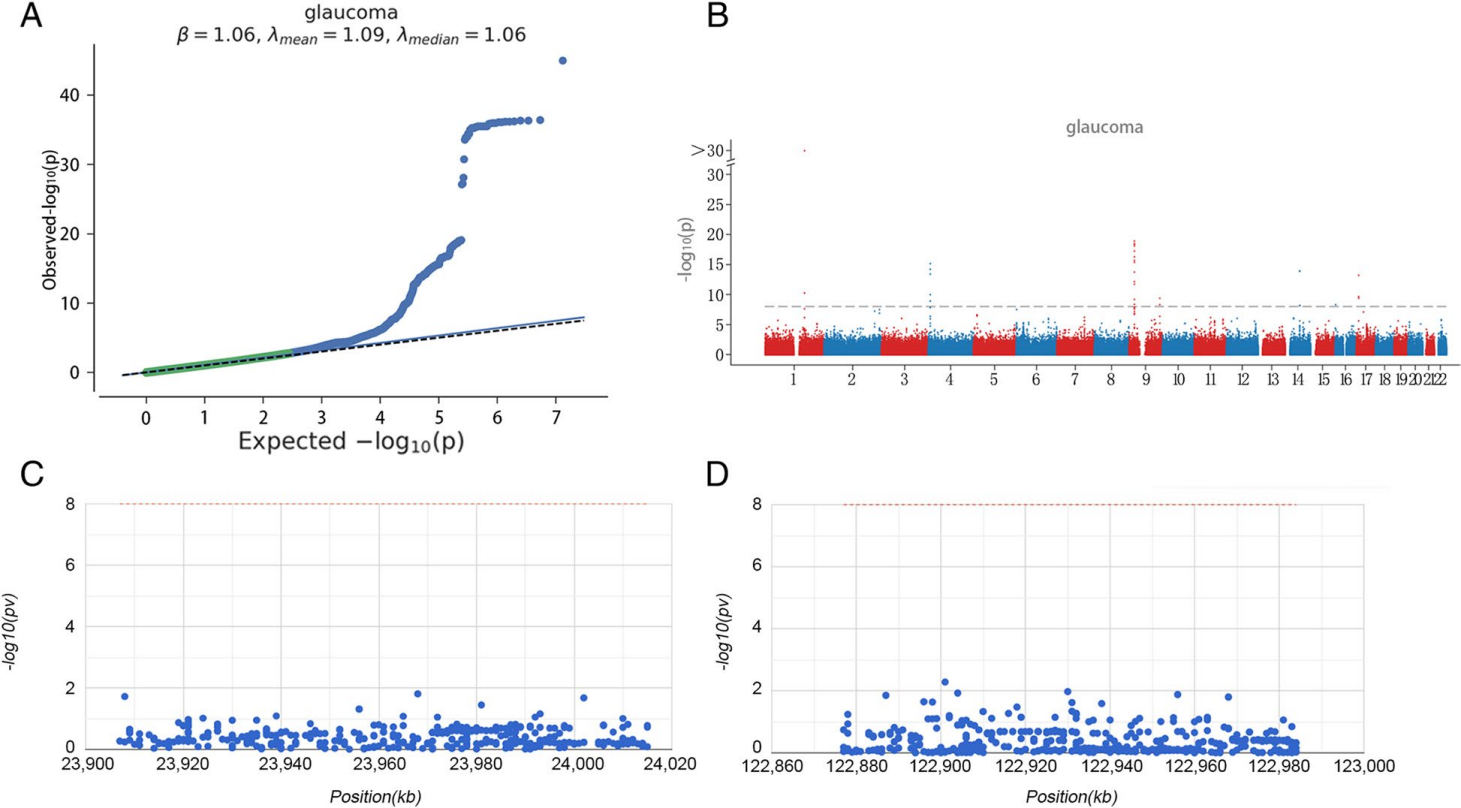
Identification of pathogenic regions of hub genes in glaucoma by GWAS analysis. **A** Q-Q plot showed SNP loci identified by GWAS data as being significantly associated with glaucoma. **B** Critical SNPs distributed in rich regions. **C**, **D** The SNP pathogenic regions corresponding to RPL15 and HSPA8

GWAS data for glaucoma were analyzed to identify the pathogenic regions of the 2 hub genes in glaucoma. Q-Q plot showed single nucleotide polymorphism (SNP) loci identified by GWAS data as being significantly associated with glaucoma (Fig. 4A). Critical SNPs distributed in rich regions were described by performing precise sites on GWAS data (Fig. 4B). The SNP pathogenic regions corresponding to RPL15 and HSPA8 were also shown, with RPL15 being located in the pathogenic region of chromosome 3 and HSPA8 being located in the pathogenic region of chromosome 11 (Fig. 4C, D). Significant SNPs corresponding to 2 hub genes were shown (Additional file 5).

### GSEA and miRNA network of hub genes

**Fig. 5:**
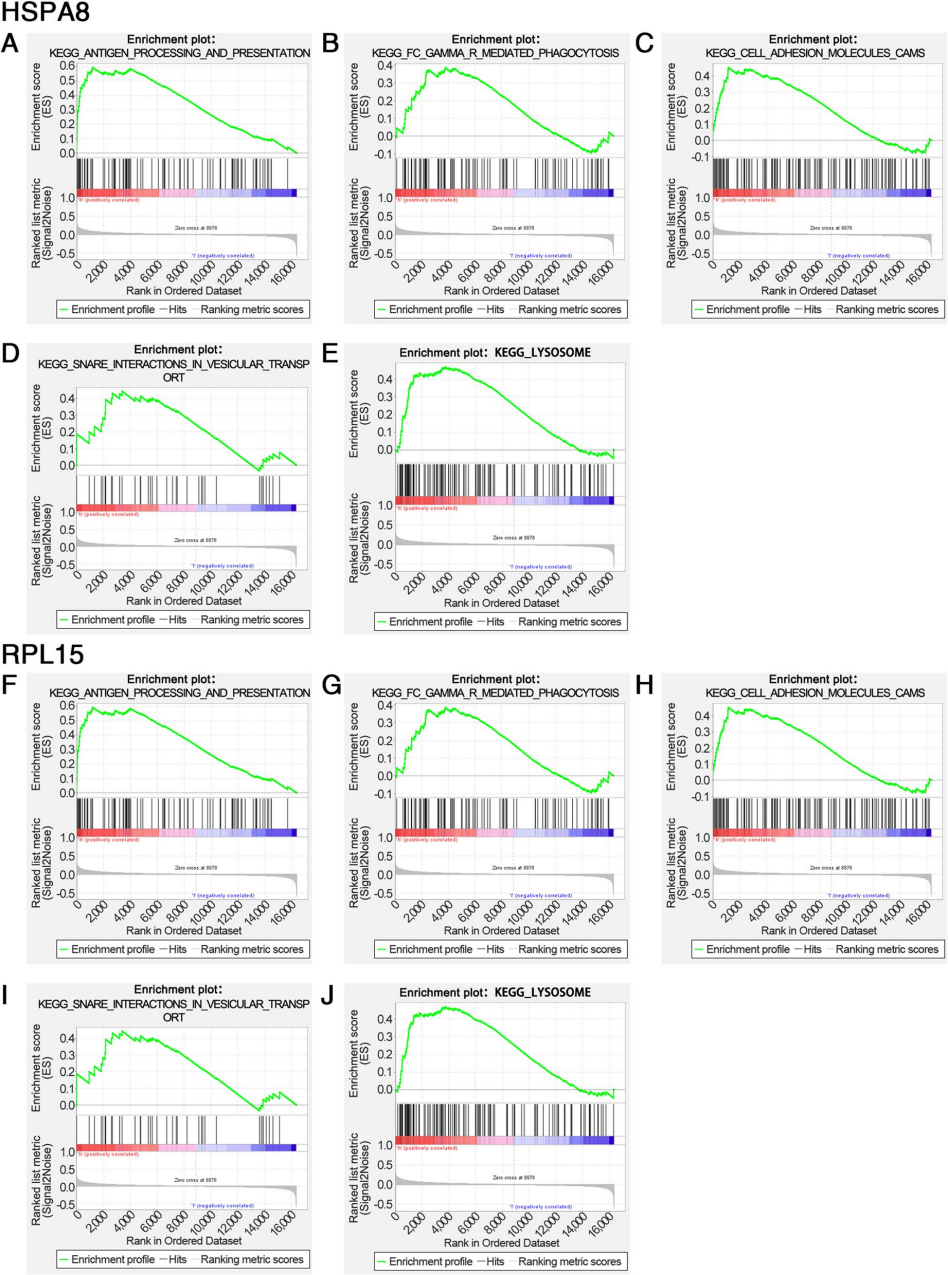
Partial GSEA results of HSPA8 and RPL15. **A-J** Highly expressed HSPA8 and RPL15 were both enriched in antigen processing and presentation, Fc gamma R-mediated phagocytosis, cell adhesion molecules, snare interactions in vesicular transport and lysosome pathways

**Fig. 6:**
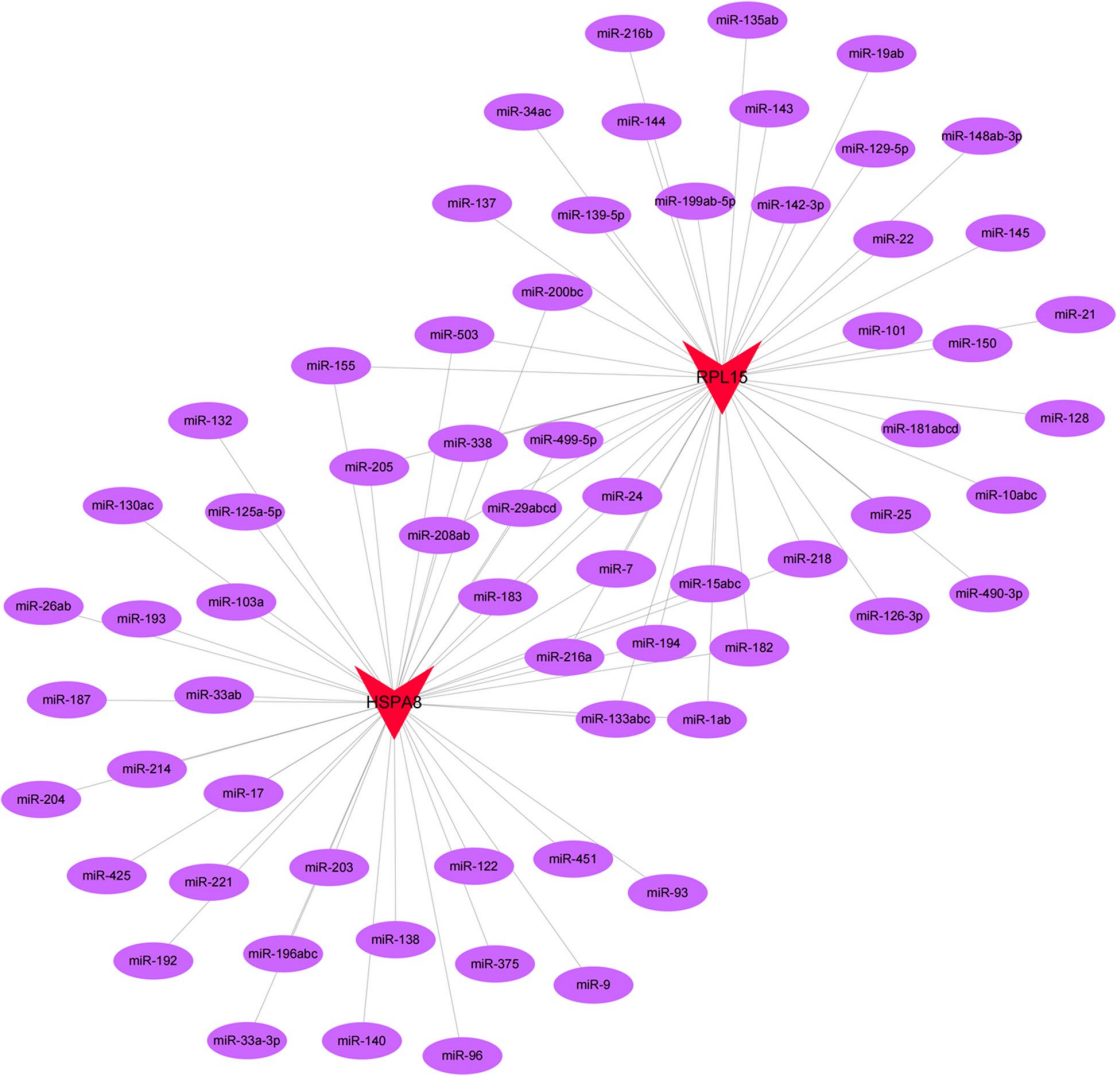
Reverse prediction miRNA network of hub genes

The specific signaling pathways involved in the 2 hub genes were further investigated to explore the potential molecular mechanisms of the hub genes affecting POAG progression. GSEA results revealed that highly expressed HSPA8 and RPL15 were both enriched in antigen processing and presentation, Fc gamma R-mediated phagocytosis, cell adhesion molecules, snare interactions in vesicular transport, lysosome, mismatch repair, RNA polymerase, protein export, steroid biosynthesis, galactose metabolism, oxidative phosphorylation and other pathways; in addition, highly expressed HSPA8 was enriched in natural killer cell mediated cytotoxicity and other pathways; highly expressed RPL15 was enriched in pentose and glucuronate interconversions and other pathways (Fig. 5A-J). Reverse prediction of the 2 hub genes was also performed through the mircode database to obtain 66 miRNAs for 84 mRNA-miRNA relationship pairs that were visualized with Cytoscape (Fig. 6).

### Motif enrichment analysis and motif-TF annotation of hub genes

**Fig. 7:**
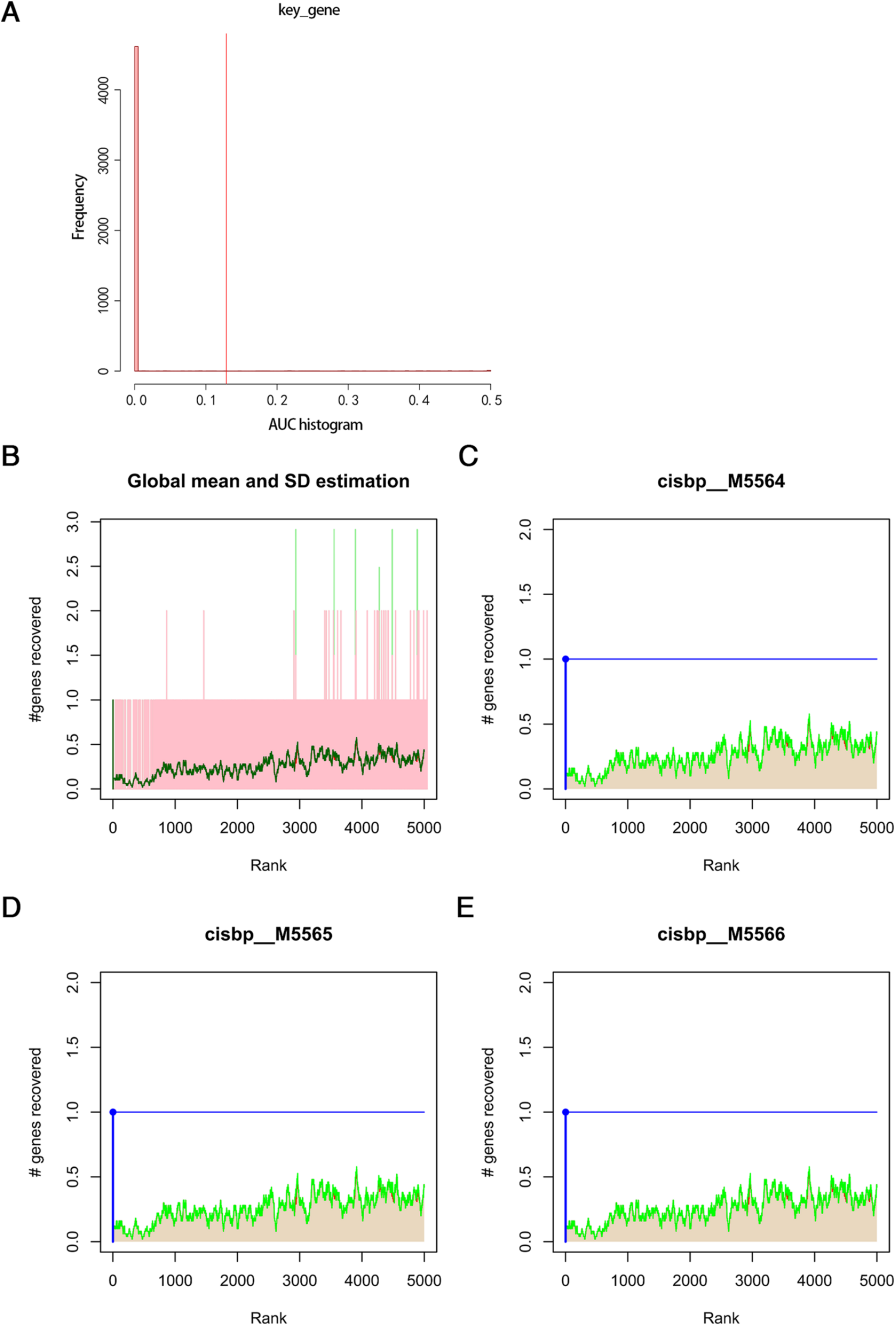
Motif enrichment analysis of hub genes. **A-E** Enrichment analysis of TFs by cumulative recovery curves. The motif with the highest NES was cisbp_M5564

The 2 hub genes were used as the gene set and analyzed in this study, and it was found that they were regulated by common mechanisms such as multiple TFs. Therefore, enrichment analysis of these TFs was performed by using cumulative recovery curves (Fig. 7A-E). The motif-TF annotation and the selection of important genes were completed. The analysis showed that the motif with the highest NES (9.69) was cisbp_M5564. Only 1 gene, HSPA8, was enriched in this motif. All motifs enriched by hub genes and corresponding TFs were shown (Table 1).

**Table 1:** Motif rank and corresponding TFs of hub genes

Rank	Motif	NES	AUC	TF_highConf	nEnrGenes	enrichedGenes
1	cisbp_M5564	9.69	0.5	HSF1 (directAnnotation).	1	HSPA8
2	cisbp_M5565	9.69	0.5	HSF1 (directAnnotation).	1	HSPA8
3	cisbp_M5566	9.69	0.5	HSF2 (directAnnotation).	1	HSPA8
4	cisbp_M2288	9.69	0.5	HSF1 (directAnnotation).	1	HSPA8
5	cisbp_M2443	9.69	0.5	HSF1; HSF2; HSF4 (inferredBy_Orthology).	1	HSPA8
6	cisbp_M6286	9.69	0.5	HSF1 (directAnnotation).	1	HSPA8
7	cisbp_M5567	9.69	0.5	HSF4 (directAnnotation).	1	HSPA8
8	cisbp_M1251	9.68	0.5		1	HSPA8
9	cisbp_M6287	9.67	0.499	HSF2 (directAnnotation).	1	HSPA8
10	cisbp_M4978	9.63	0.497	NR5A1 (inferredBy_Orthology).	1	HSPA8

### Correlation analysis between hub genes and glaucoma-related pathogenic genes

**Fig. 8:**
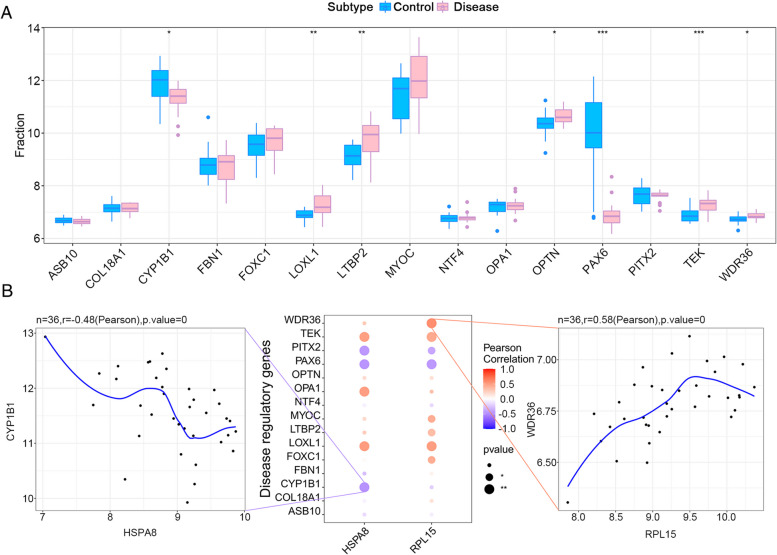
Correlation analysis between hub genes and glaucoma-related pathogenic genes. **A** Differential expression analysis of causative genes. The expression levels of CYP1B1, LOXL1, LTBP2, OPTN, PAX6, TEK and WDR36 were different between the normal groups and the POAG groups. **B** HSPA8 was significantly negatively correlated with CYP1B1 (Pearson r = − 0.48) and RPL15 was significantly positively correlated with WDR36 (Pearson r = 0.58)

A total of 5011 causative genes associated with glaucoma were obtained from the GeneCards database. The expression levels of 2 hub genes and the top 20 of causative genes were analyzed. The expression levels of CYP1B1, LOXL1, LTBP2, OPTN, PAX6, TEK and WDR36 were found to be different between the normal groups and the POAG groups (Fig. 8A). Next, correlation analysis between the hub genes and the causative genes in glaucoma was performed. The expression levels of hub genes were significantly correlated with the expression levels of multiple glaucoma-related genes, among which HSPA8 was significantly negatively correlated with CYP1B1 (Pearson r = − 0.48) and RPL15 was significantly positively correlated with WDR36 (Pearson r = 0.58) (Fig. 8B).

### CMap drug prediction of POAG

**Fig. 9:**
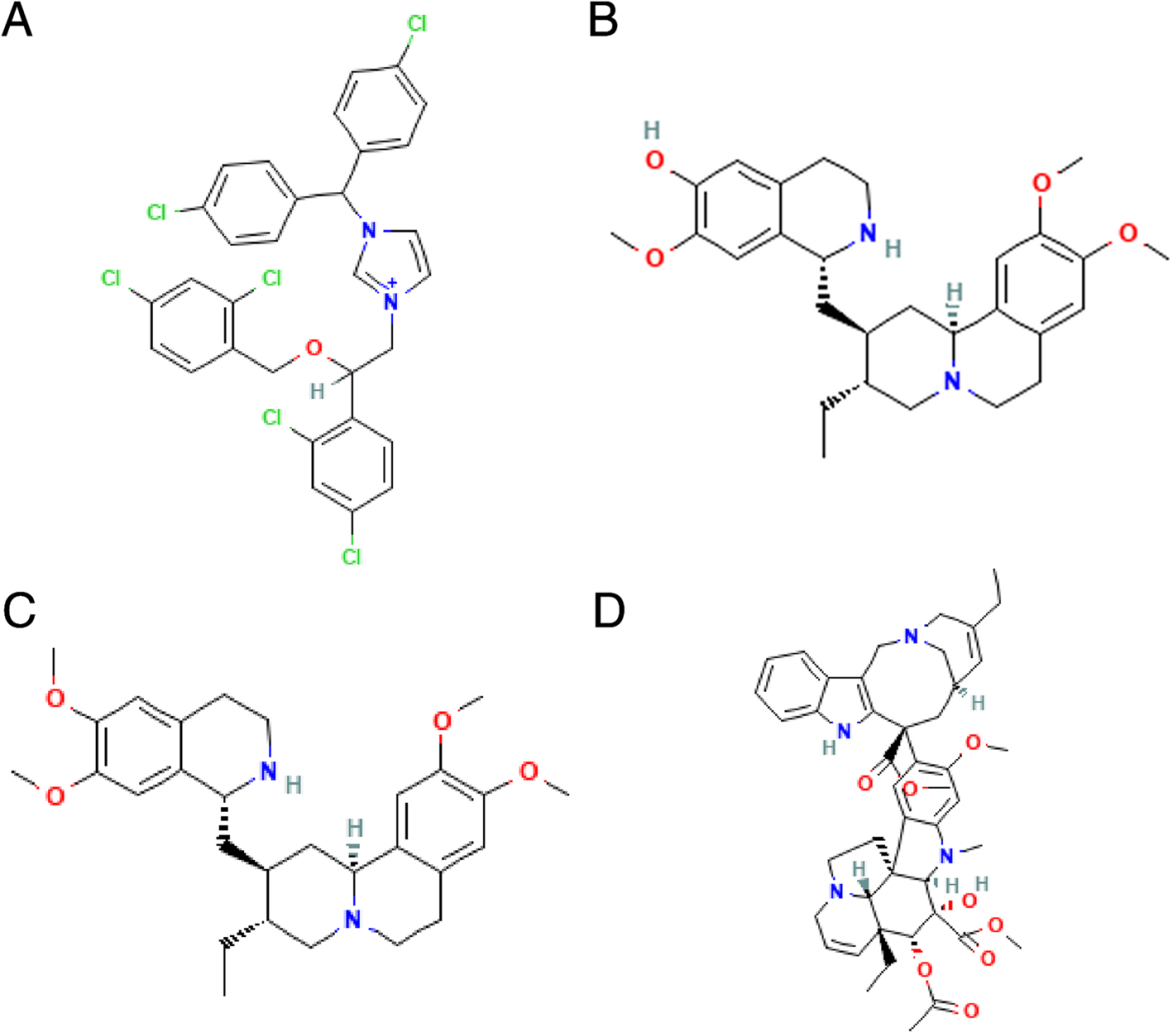
CMap drug prediction. **A-D** Molecular structure of calmidazolium, cephaeline, emetine and vinorelbine

What’s more, drug prediction on TOP150 up-regulated genes and TOP150 down-regulated genes of the GSE27276 dataset was performed by CMap database. The results revealed that the expression profiles of drug disturbances such as calmidazolium, cephaeline, emetine and vinorelbine were most significantly negatively correlated with the expression profiles of POAG disturbances, which indicated that these drugs could alleviate or even reverse the POAG disease state (Fig. 9A-D).

### IOP in COH glaucoma model group and normal control group

**Fig. 10:**
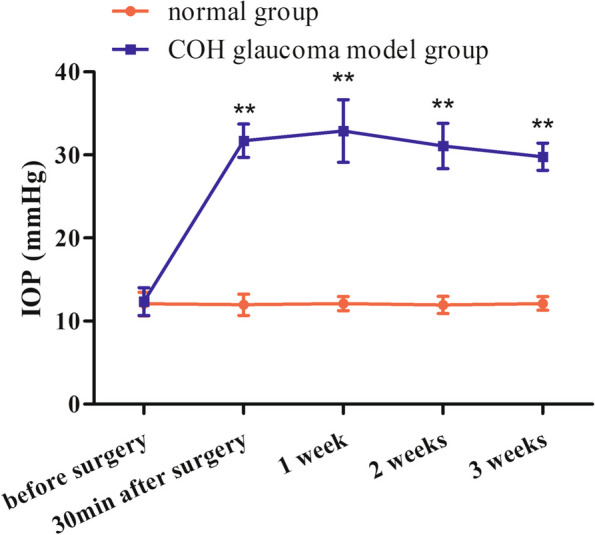
Changes of IOP with time in the normal group and the COH glaucoma model group

The results illustrated that there were no significant differences in IOP between the 2 groups before surgery, which indicated that the groups were comparable. However, there were significant differences in IOP between the 2 groups at 30 minutes, 1 week, 2 weeks and 3 weeks after surgery, and IOP was higher in the COH glaucoma model group than in the normal control group. There were no significant differences in IOP between all-time points of the normal control group. However, the IOP at each time point after surgery was higher than that before surgery in the COH glaucoma model group, and the differences were statistically significant, which suggested that the glaucoma model of COH rats was successfully established (Table 2; Fig. 10).

**Table 2:** Changes of IOP with time in two groups (mean ± standard error)

Group	n	IOP (mmHg)				
		Before surgery	30 min after surgery	1 week	2 weeks	3 weeks
Normal group	4	12.07 ± 1.41	11.97 ± 1.29	12.10 ± 0.86	11.95 ± 1.03	12.13 ± 0.81
COH glaucoma model group	4	12.35 ± 1.68	31.72 ± 2.00^*†^	32.89 ± 3.76^*†^	31.08 ± 2.74^*†^	29.79 ± 1.63^*†^

^*^P < 0.05 vs before surgery; ^†^*P* < 0.05 vs normal group

### Validation of hub genes in glaucoma model of COH rats

**Fig. 11:**
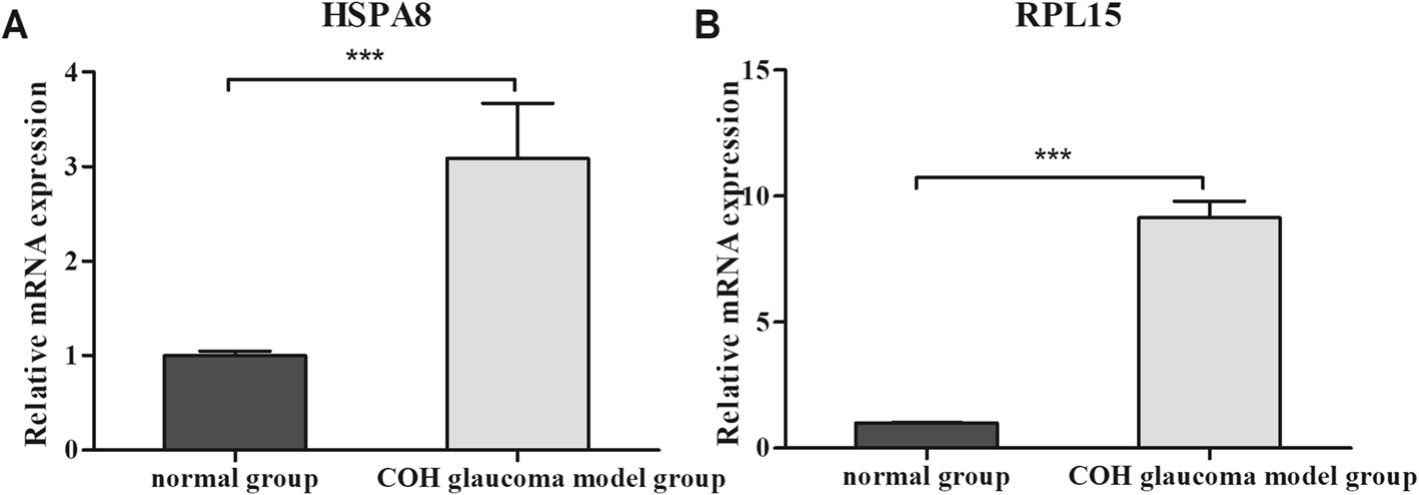
The mRNA level of hub genes in rat models. **A** Expression of Hspa8. **B** Expression of Rpl15. ∗∗∗*P* < 0.001

In this study, the simulated POAG model of COH rats was induced by episcleral vein cauterization to verify the function of hub genes in DEARGs. It is due to following reasons. Firstly, the ocular anatomy of rodents such as rats is like that of humans, and pressure-dependent models of rats can produce lesions like glaucoma in humans. Therefore, rat COH models have been widely used in glaucoma research [25]. Secondly, studies have shown that autophagy occurring in ocular tissues such as TM and RGCs is associated with POAG [16, 26]. Autophagy is found to be activated, autolysosomes and autophagy associated markers are obviously detected in the retina and the optic nerve, and autophagic flux is increased in the COH glaucoma model, which indicates that this animal model is suitable for autophagy research [18, 26, 27]. According to qRT-PCR results, the expression levels of Hspa8 and Rpl15 in the ocular tissues were higher in the COH glaucoma model group than that in the normal control group, and the differences were statistically significant, which was consistent with the results of bioinformatics analysis above (Fig. 11A, B). These results suggested that HSPA8, RPL15 and their downstream signaling pathways may be involved in the progression of POAG by regulating ocular autophagy.

## Discussion

Due to the lack of evidence, the etiology of POAG is not yet fully clarified. According to previous studies, POAG is perhaps the result of the interaction of multiple genetic and environmental factors [28]. Risk factors for POAG include ocular hypertension, advanced age, female sex, family history of glaucoma, refractive error, and steroid use. Since IOP is currently the only known modifiable risk factor, the treatment for POAG focuses on lowering IOP by medical or surgical l means [29, 30]. Successful glaucoma treatment was once defined as a reduction in IOP to within 2 standard deviations of the normal population mean. However, in some cases, RGC degeneration and visual loss continued to occur in patients despite normal or well-controlled IOP, and clinicians are now trying to find other viable treatments to prevent progression of visual impairment and preserve each patient ‘s visual field [31–33]. Therefore, the pathogenesis of POAG need to be further explored.

The characteristic of POAG is that the angle of the anterior chamber is open, and drainage of aqueous humor is often blocked in the TM-Schlemm’s canal system. Histological and physiological observations suggest that pathological changes such as narrowing, occlusion or sclerosis of Schlemm’s canal and TM occur in the anterior segment of POAG patients, which may be due to deposition of extracellular matrix, decrease of cell number, and accumulation of fibrous components [14, 32, 34, 35]. The causes of TM system structural and functional disorders include inflammation, cell aging, oxidative damage, and decreased expression of cell markers [32]. The posterior segment lesions of POAG are characterized by typical depressed atrophy of the optic nerve head and the chronic progressive loss of RGCs. Explanations for RGCs loss include neurotrophic factor deprivation, inflammation, mitochondrial dysfunction, ischemia, oxidative stress, glutamate excitotoxicity and glial activation [15, 18, 33]. In conclusion, changes in the anterior and posterior segments of POAG may involve a variety of pathogenesis. Increasing evidence indicates that autophagy, as a major physiological metabolic pathway for the degradation cycle of misfolded proteins or damaged organelles, as well as a basic cell survival mechanism against various stressors, may be involved in the pathogenesis of POAG [36–38].

Whether autophagy has a beneficial or detrimental effect on POAG is controversial. Activation of autophagy contributes to removing damaged components and inhibiting cell death under normal physiological conditions [39]. Autophagy is cytoprotective for RGCs, genetic down-regulation of autophagy reduces RGC survival after optic nerve injury, while pharmacological up-regulation of autophagy decreases RGC loss and oxidative damage. For instance, rapamycin exerts neuroprotection by enhancing autophagy [17, 27, 40]. Optic nerve degeneration in POAG may be associated with autophagic flux impairment [41]. Autophagy regulation is expected to become a new therapeutic strategy for irreversible retinal neurodegenerative diseases such as POAG. Like neurons such as RGCs, TM cells that are not capable of self-renewal require increased turnover of proteins and organelles through autophagy to maintain cellular homeostasis and adapt to mechanical stress, which suggests that autophagy also plays a protective role in TM cells [42]. In addition, impaired autophagic flux has been observed in an in vitro model of aging, which may be one of the factors leading to the progressive functional failure of TM cells with age [16]. Therefore, progressive TM dysfunction is related to reduced autophagy, which contributes to the exploration of POAG pathogenesis. However, dysregulation of autophagy may progress to autophagic programmed cell death or trigger cell death through crosstalk with apoptosis pathways if the stress exceeds physiological tolerance or under pathological conditions [12, 43, 44]. Park showed that the neuroprotective attempt of autophagy in early stage failed owing to sustained and chronic IOP elevation, excessive autophagy induced apoptosis and autophagic cell death of RGCs, while inhibition of autophagy by 3-methyladenine treatment could promote RGC survival, which indicated that inhibition of excessive autophagy might prevent RGC degeneration and death in POAG [18, 45]. These studies indicated that insufficient or excessive autophagy affected POAG, and appropriate activation of autophagy facilitated the protection of RGCs and TM cells. In contrary, excessive autophagy caused cell death. However, how to define the appropriate activation range of autophagy and how to precisely regulate it still need to be further explored.

In this study, 67 DEARGs potentially associated with POAG, including 30 up-regulated genes and 37 down-regulated genes, were identified based on the intersection of DEGs in GSE27276 dataset and autophagy-related genes in GeneCards database by bioinformatics analysis. To elucidate the underlying molecular mechanisms of 67 DEARGs, their biological functions were evaluated by GO and KEGG enrichment analysis. The results revealed that DEARGs were mainly enriched in pathways such as regulation of proteolysis, regulation of inflammatory response, astrocyte development, regulation of proteasomal protein catabolic process, regulation of protein kinase B signaling, regulation of autophagy, and response to glucocorticoid. The correlation between these pathways and autophagy or glaucoma can also be supported by previous studies. Astrocytes with impaired autophagy cause neurodegenerative changes in glaucoma by affecting RGCs [46]. Protein kinase B is also known as AKT, and the activation of PI3K/AKT signaling inhibits the autophagy pathway and reduces RGC apoptosis in the development of glaucoma [47]. Long-term glucocorticoid treatment reduces the activity of autophagy, and TM cell dysregulation of homeostasis and function eventually forms features like POAG [48]. Subsequently, the PPI network of DEARGs was constructed using the STRING database and 5 key genes were selected according to MCODE, including UBB, RPL15, HSPA8, RPS5 and PSMC1. These 5 key genes were validated in the GSE138125 dataset, and 2 hub genes, HSPA8 and RPL15, were finally screened out.

The role of the 2 hub genes in POAG has been rarely reported in the previously published studies so far. Heat shock 70-kDa protein 8 (HSPA8, also known as HSC70) is encoded by the HSPA8 gene and acts as a cytosolic molecular chaperone that involves in multiple balancing mechanisms, including chaperone-mediated autophagy, degradation of misfolded proteins, and dilution of toxic intracellular components [49, 50]. High expression of HSPA8 has been identified in various cancer cells and is involved in the growth and autophagy regulation of cancer cells [51]. Decreased expression of HSPA8 may lead to the accumulation of neurodegenerative disease-related proteins and induce diseases such as Parkinson’s disease (PD) and Alzheimer’s disease (AD), which suggests that HSPA8 is important for neuroprotection [52–54]. Heat shock protein (HSP) synthesis increases in response to multiple forms of metabolic stress to exert protective functions, and the most studied retinal HSPs are members of the 70-kDa heat shock protein (HSP70) family, mainly including HSPA8 (the constitutive form) and HSP72 (the inducible protein), which are rapidly induced in the mammalian retina when being subjected to stressors [55, 56]. Previous studies demonstrated that elevated IOP caused increased expression of HSP72, while the expression of HSPA8 had no significant differences in the rat COH glaucoma model created by trabecular laser photocoagulation [57]. However, in this study, it was found that the expression of Hspa8 was up-regulated in the rat COH glaucoma model that was induced by episcleral vein cauterization, compared with the normal control group, which confirmed the results of bioinformatics analysis of POAG to some extent. In addition, the expression of HSP60 and HSP27 was increased in POAG patients’ eyes compared with normal human eyes [55]. These results suggested that HSPs may play a role as a cellular defense mechanism against glaucoma stress or injury [56]. It has been demonstrated that inducing the expression of HSP70 family, such as HSP72, can improve the survival of RGC under harmful conditions and reduce optic nerve injury in the rat glaucoma model and optic nerve crush model [57–60]. Inducing HSPA8 expression to protect RGC may be a novel strategy for endogenous neuroprotective therapy of POAG, which remains to be verified by further experiments.

RPL15 encodes for the protein 60S ribosomal L15 that plays a key role in protein synthesis, assembly of ribosomal subunits and processing of rRNA. Alterations in RPL15 expression may affect the efficiency of ribosomal translation [61, 62]. RPL15 gene deletion was identified in Diamond-Blackfan anemia [63]. The expression of RPL15 varied with the type of cancer, and it was highly expressed in esophageal cancer, gastric cancer, hepatocellular carcinoma, and colon cancer, but lowly expressed in cutaneous squamous cell carcinoma and pancreatic ductal adenocarcinoma [61, 64–68]. The expression of RPL15 was closely related to several neurodegenerative diseases, such as AD and PD [69, 70]. RPL15 could regulate the RP-MDM2-p53 signaling pathway and p53 was involved in a variety of stress responses. RPL15 knockdown inhibited p53 degradation through ribosomal stress, and increased stability and transcriptional activity of p53 to mediate cell cycle arrest and apoptotic cell death, which might be the mechanism, by which RPL15 knockdown promoted the apoptosis of hepatocellular carcinoma, colon cancer and leukemia cells through the intrinsic pathway of mitochondria [61, 64, 71]. In addition, p53 could also regulate autophagy that was activated through inhibiting mTOR by p53 under stressor stimulation [72]. RPL15 knockdown was found to induce autophagic cell death in human leukemia cells, perhaps also due to the regulatory effect of RPL15 on p53 [71]. It has been shown that the stress response pathway of p53 may be a potential risk factor for POAG [73]. Apoptosis of TM cells and RGCs in POAG involved upregulation of p53, while downregulation of p53 favored protection of TM and retina [74–76], which suggested that RPL15 that was involved in p53 regulation may also be associated with POAG. In this study, the results of bioinformatics analysis indicated that the expression of RPL15 was higher in the TM of POAG patients than that of normal controls, which was verified in subsequent animal experiments. Whether RPL15 can be a new therapeutic target for POAG and whether the specific mechanism of RPL15 can affect POAG need to be further investigated.

The development of POAG is closely related to immune infiltration, while no systematic studies elucidated the functional link between autophagy and immune infiltration in POAG. Therefore, to further explore the mechanism by which HSPA8 and RPL15 affect POAG progression, immune cell infiltration analysis was performed. Plasma cells and Dendritic cells activated were significantly lower in the POAG group compared with the normal group. Meanwhile, HSPA8 was positively correlated with Eosinophils and T cells CD4 memory resting, while negatively correlated with B cells naive and T cells CD4 naive; RPL15 was positively correlated with Dendritic cells resting, T cells CD4 memory resting, Macrophages M2 and Eosinophils, while negatively correlated with B cells naive, Dendritic cells activated and T cells CD4 naive. These results suggested that immune factors may be involved in the pathogenesis of POAG, and hub genes were closely related to the level of immune cell infiltration. Previous studies have also illustrated that HSPA8 can serve as a molecular switch regulating the endocytic and autophagic pathways and plays a central role at different critical steps of antigen presentation to CD4 T cells during stress [77, 78]. HSPs stimulated T cell proliferation, and it was thought to be the cause of progressive neurodegeneration, and immunoregulatory deficiency led to neuroinflammatory response in POAG patients, which suggested that this might be the mechanism of HSPA8 affecting POAG through immune pathway [79]. RPL15 knockdown increased the cytotoxic T lymphocytes and decreased the T-regulatory cells to enhance anti-tumor immune response [80]. However, whether RPL15 has an immune effect on POAG has not been studied.

Next, the GWAS analysis was conducted to confirm that the pathogenic regions of HSPA8 and RPL15 in glaucoma were located on chromosome 11 and 3, respectively, and significant SNPs corresponding to 2 hub genes were found. To further investigate the specific signaling pathways involved in the 2 hub genes, GSEA was performed, and the results showed that highly expressed HSPA8 and RPL15 shared many enriched pathways. Among them, immune-related pathways included antigen processing and presentation, Fc gamma R-mediated phagocytosis, and cell adhesion molecules, which verified that the 2 hub genes may affect POAG through the immune pathway. Autophagy-related pathways contained snare interactions in vesicular transport and lysosome, which suggested that the 2 hub genes may be involved in POAG progression by affecting autophagy. Furthermore, the highly expressed HSPA8 and RPL15 were also enriched in gene expression and energy metabolism pathways such as mismatch repair, RNA polymerase, protein export, steroid biosynthesis, galactose metabolism and oxidative phosphorylation. Afterwards, the miRNA network of the 2 hub genes was predicted, and the motifs enriched by the hub genes and corresponding TFs were analyzed to gain insight into the expression regulation process of the hub genes from both post-transcriptional and transcriptional initiation aspects. The results suggested that HSPA8 may be regulated by TFs such as HSF1 and HSF2, which were consistent with previous studies. In addition, previous studies have also found that both HSF1 and HSF2 are expressed at relatively high levels in rat RGCs [81]. How HSFs are activated to regulate HSPA8 and how they affect POAG need more investigations.

To further explore the relationship between hub genes and POAG, correlation analysis between hub genes and glaucoma pathogenic genes was performed. The results revealed that HSPA8 was significantly negatively correlated with CYP1B1, and RPL15 was significantly positively correlated with WDR36. WDR36 has been reported as a causative gene for POAG [82]. CYP1B1 was first identified as a causative gene of primary congenital glaucoma, and CYP1B1 mutations have also been identified in POAG [83]. Next, the DEGs in GSE27276 dataset were used to predict the targeted therapeutic drugs for POAG. The results proved that the expression profiles of drug disturbances such as Calmidazolium, Cephaeline, Emetine and Vinorelbine were most significantly negatively correlated with the expression profiles of disease disturbances. Calmidazolium is a calmodulin inhibitor [84]. Both Cephaeline and Emetine are pleiotropic alkaloids isolated from ipecacuanha trees, and the difference is that Emetine contains a methoxy group while Cephaeline contains a hydroxyl group. On the other hand, Cephaeline is better tolerated by patients than Emetine [85, 86]. Vinorelbine is a tubulin inhibitor [87]. Our study suggested that these drugs had the potential to alleviate or even reverse POAG.

This study still has some limitations. First of all, we obtained a series of theoretical results by bioinformatics analysis of previously published datasets. The specific mechanisms, pathogenic loci, signaling pathways and regulatory processes of hub genes affecting POAG still need to design more precise experiments for research. Secondly, the human TM tissues were used for bioinformatics analysis in this study, and due to limitations in the animal model verification, the whole eyeball tissues of rats were used for qRT-PCR. Although consistent results were finally obtained, TM tissues or retinal tissues can be isolated separately for more accurate experiments in the future to identify the specific pathogenic sites of POAG by hub genes. Thirdly, due to the small number of animal experimental samples used for hub genes validation, animal experiments with larger sample sizes or animal species and surgery methods closer to the pathological changes of POAG in humans are needed to prepare for future clinical studies of novel molecular markers.

## Conclusions

In summary, 67 potential DEARGs from the POAG-related dataset of the GEO database were identified, and 2 hub genes, HSPA8 and RPL15 were selected by constructing the PPI network and using another dataset for validation. To further investigate the mechanism by which hub genes influence POAG, a series of analyses were performed using bioinformatics tools. Finally, animal experiments were carried out to preliminarily verify that HSPA8 and RPL15 may affect the development of POAG by regulating autophagy. Further experiments are needed in the future to explore the specific mechanisms by which 2 hub genes regulate autophagy in POAG. Similarly, more studies are needed to demonstrate whether the autophagic process is a neuroprotective mechanism for POAG or a pathway to induce cell death. However, whether protection or injury, targeted regulation of autophagy by molecular markers provides us with new ideas for the treatment of POAG in clinical practice.

### Supplementary Information

**Additional file 1.**

**Additional file 2.**

**Additional file 3.**

**Additional file 4.**

**Additional file 5.**

## Data Availability

The datasets used and/or analysed during the current study are available from the Gene Expression Omnibus database (GEO: GSE27276 and GSE138125) https://www.ncbi.nlm.nih.gov/geo/, the GeneCards database https://www.genecards.org/, the Metascape database https://metascape.org/, and the Gene Atlas database http://geneatlas.roslin.ed.ac.uk/.

## Abbreviations

POAG: Primary open-angle glaucoma
GEO: Gene Expression Omnibus
DEARGs: Differentially expressed autophagy-related genes
PPI: Protein-protein interaction
GWAS: Genome-wide association study
GSEA: Gene set enrichment analysis
qRT-PCR: Quantitative real time polymerase chain reaction
RGC: Retinal ganglion cell
TM: Trabecular meshwork
DEGs: Differentially expressed genes
GO: Gene Ontology
KEGG: Kyoto Encyclopedia of Genes and Genomes
COH: Chronic ocular hypertension
TFs: Transcription factors
NES: Normalized enrichment score
AUC: Area under the curve
CMap: Connectivity map
SPF: Specific-pathogen free
SD: Sprague-Dawley
IOP: Intraocular pressure
SNP: Single nucleotide polymorphism
HSPA8: Heat shock 70-kDa protein 8
PD: Parkinson’s disease
AD: Alzheimer’s disease
HSP: Heat shock protein
HSP70: 70-kDa heat shock protein
